# Management of children consulting a specialized psychotraumatology unit in Tunisia: About 66 patients

**DOI:** 10.1192/j.eurpsy.2023.1215

**Published:** 2023-07-19

**Authors:** R. Gadhoum, S. Bourgou, H. Ben Youssef, H. Rezgui, A. Ben hamouda, M. Daoued, F. charfi, A. belhadj

**Affiliations:** Child and Adolescents psychiatry, Mongi Slim Hospital, Tunis, Tunisia

## Abstract

**Introduction:**

Children exposed to trauma present particular clinical features, therefore this population requires specific care and support.

**Objectives:**

Study the clinical features and care modalities of children consulting Trauma & Resilience Unit.

**Methods:**

It is a retrospective descriptive study of children consulting Trauma & Resilience unit at the child psychiatry department of Mongi Slim Hospital in Tunis (Tunisia) between January and April 2022. We collected data concerning the course of clinical features and care modalities with help of an exploitation form. Statistical analyses were performed by SPSS26.

**Results:**

Our study included 66 patients. The sex ratio was 1. The mean age was 10.46 ± 3.24 years. The main symptoms initially presented were hypervigilance in preschoolers (p=0.02), avoidance behaviors in school-age children (p<0.05) and flashbacks in adolescents (p<0.05).

The diagnosis of adjustment disorders was made in 38.4% of the cases of which 32% were victims of physical assault. Post-traumatic stress disorder was diagnosed in 25.7% of cases, 35.2% of which were victims of sexual assault. A normal psychiatric examination was significantly frequent in cases of psychological assault (p=0.04). The Child Protection Officer was alerted in 46.2% of cases. The school was notified of the repercussions on children health in 38.4 % of cases. Psychotherapy was provided in 86.2% of cases. We prescribed pharmacological treatment for 14% of patients with 59% antidepressants in 59% and sleep medication in 41%.

**Conclusions:**

Management of children in psychotraumatology units turns out to be challenging. Therefore, working on the links between the various partners involved, while respecting the differences and specificities of each, is an essential prognostic element for the children and adolescents concerned.

**Disclosure of Interest:**

None Declared

